# ﻿*Petrocodonwui* (Gesneriaceae), a new species from Guizhou, China

**DOI:** 10.3897/phytokeys.225.99660

**Published:** 2023-04-19

**Authors:** Ren-Bo Zhang, Tan Deng, Nan Li, Fang Wen

**Affiliations:** 1 Department of Biology, Zunyi Normal College, Zunyi, CN-563000 Guizhou, China Zunyi Normal College Zunyi China; 2 Guangxi Key Laboratory of Plant Conservation and Restoration Ecology in Karst Terrain, Guangxi Institute of Botany, Guangxi Zhuang Autonomous Region and Chinese Academy of Sciences, CN-541006 Guilin, Guangxi, China Guangxi Institute of Botany, Guangxi Zhuang Autonomous Region and Chinese Academy of Sciences Guilin China; 3 Gesneriad Committee of China Wild Plant Conservation Association (GC), National Gesneriaceae Germplasm Resources Bank of GXIB (NGGRB), Gesneriad Conservation Center of China (GCCC), CN-541006 Guilin, Guangxi, China National Gesneriaceae Germplasm Resources Bank of GXIB (NGGRB), Gesneriad Conservation Center of China (GCCC) Guilin China

**Keywords:** Didymocarpoideae, flora of Guizhou, lithophyte, new taxon, *Petrocodonchishuiensis*, taxonomy

## Abstract

*Petrocodonwui* F.Wen & R.B.Zhang (Gesneriaceae), a typically lithophyte occurring in the Danxia areas of north-western Guizhou, China, is described and illustrated as new to science. The new species shows overall similarity with *P.chishuiensis* Z.B.Xin, F.Wen & S.B.Zhou, which is also its sister species, based on molecular evidence. The new species can be distinguished from *P.chishuiensis* by the elongated rhizome, the relatively long indumentum on the peduncle, the shape, size and indumentum of calyx lobes, the location of the stamens in the corolla tube and the shape, size and indumentum of the stigma. We provide a diagnosis, detailed description, photographic images and a table with taxonomic notes to distinguish several other morphologically similar *Petrocodon* species.

## ﻿Introduction

The genus, *Petrocodon* Hance (subfamily Didymocarpoideae, family Gesneriaceae), has 47 species, including the newly-published taxon *P.asterostriatus* F.Wen, Y.G.Wei & W.C.Chou (Möller 2019; GRC 2022; Yang et al. 2022). All known species of this genus are lithophytes (= rock dwelling), either growing in karst topographies or in Danxia landforms. This genus is mainly distributed in karst areas from eastern and south-western China to the northern Indo-China Peninsula, especially in Guangxi of China and North Vietnam (Wei 2018; Wei et al. 2022). Undoubtedly, China is the biodiversity centre of *Petrocodon* because there are at least 44 species (especially in Guangxi, with 25 species) (Wei 2018). Although many new taxa have been discovered and published in recent years, there were only two new taxa discovered and confirmed from Danxia landforms, namely *P.asterocalyx* F.Wen, Y.G.Wei & R.L.Zhang (Zhang et al. 2018) and *P.chishuiensis* Z.B.Xin, F.Wen & S.B.Zhou (Xin et al. 2020). It is well known that all species of *Petrocodon* are typically lithophilous and usually segregated into unique habitats, for example, karst caves and Danxia gorges, so that most of the species in this genus are narrow endemics (Fu et al. 2022a).

In early August 2021, we conducted a plant diversity survey in Xishui National Nature Reserve in Guizhou Province, China. We noticed an unknown species of Gesneriaceae growing on the surface of Danxia cliff in Niuqingshan, Dabaitang of the Xishui National Reserve. Based on its lithophytic habit and taxonomical characters, we considered it might belong to the genus *Petrocodon*. Upon closer examination of the flowering specimens in the lab and careful observation of living plants for comparison of vegetative and reproductive organs, we soon discovered several noticeable morphological differences that do not match any known *Petrocodon* species. Moreover, only two known species of *Petrocodon* endemic to Danxia landforms were confirmed before this species was discovered. Using morphology or molecular evidence, the new taxon of *Petrocodon* is recovered as sister to *P.chishuiensis*, but remarkably different from other species in surrounding cities and counties by some obvious characters. Thus, we concluded it corresponds to a species new to science.

## ﻿Materials and methods

### ﻿Taxonomic revision

The studied specimens were collected from the type locality and deposited in the Guangxi Institute of Botany Herbarium (IBK) and the Botany Herbarium of Zunyi Normal College (**ZY**). The macromorphological features were observed on the specimen sheets and taken from field notes and reports from the conservation nurseries at the National Gesneriaceae Germplasm Resources Bank (**NGGB**) of the Guangxi Institute of Botany (**GXIB**) and the Gesneriad Conservation Center of China (**GCCC**). Micromorphological observations were analysed and photographed using a stereomicroscope (Olympus Optical Microscope CX23). The morphological characters were compared with the protologue and type specimens of previously described *Petrocodon* species (Wang et al. 1990, 1998; Wei et al. 2010; Wei 2018), in particular those involving new taxa of *Petrocodon* from Guangxi and adjacent provinces (see notes) and herbarium specimens deposited at relevant herbaria (e.g. HITBC, IBK, IBSC, KUN, PE and VMNM).

The description of the new species follows the terminology used by Wang et al. (1998) and Harris and Harris (2001). Assessment of the conservation status of the new species was made according to the Categories and Criteria of the IUCN (IUCN Standards and Petitions Committee 2022).

### ﻿Phylogenetic analysis

Leaf material of the undescribed species was collected from the type locality in Xishui County (Guizhou, China) and immediately dried in silica gel for DNA extraction (Chase and Hills 1991). The nuclear ribosomal internal transcribed spacer (ITS) region and plastid *trnL-F* intron spacer region (*trnL-F*) were used in the study. Primers, DNA extraction, PCR amplification and sequencing followed Yang et al. (2022). To elucidate the phylogenetic affinities of the undescribed species within the genus, we incorporated 39 samples representing 25 species (Table 1), following Yang et al. (2022). The ingroup contained 37 samples from 23 species of *Petrocodon*. *Primulinadryas* (Dunn) Mich. Möller & A.Weber and *P.pinnata* (W.T.Wang) Yin Z. Wang were chosen as outgroups, based on previous phylogenetic analyses (Möller et al. 2009; Weber et al. 2011; Zhang et al. 2018). We performed phylogenetic analyses of the included *Petrocodon* species, based on the combined dataset of *trnL-F* and ITS sequences using Maximum Likelihood (ML). We employed IQ-TREE v.2.0.6 (Nguyen et al. 2015) with 1000 bootstrap replicates (Hoang et al. 2018) and default ModelFinder (Kalyaanamoorthy et al. 2017) and found K3Pu+F+G4 as the best fit substitution model. Tree visualisation was carried out in FigTree v.1.4.3 (http://tree.bio.ed.ac.uk/software/figtree/). Visual comparison of optimal tree topologies of *trnL-F* and ITS datasets was used to compare topological inconsistencies. Conflicts between tree topologies were considered significant when the inconsistent topologies received bootstrap values ≥ 80% (Fu et al. 2022b). As visual inspection showed no significant topological contradictions for bootstrap support consistency between the *trnL-F* and ITS datasets (results not shown), the two regions were combined in further analyses.

**Table 1.** The voucher and GenBank accession numbers used in this study.

Species name	Voucher number	*trnL-F*	ITS
*Primulinadryas*	C7a	FJ501524	FJ501348
*Primulinapinnata*	G26	FJ501526	FJ501349
*Petrocodonretroflexus*	–	KX579061	KX579060
*Petrocodonnivelolanosus*	–	JF697588	JF697576
*Petrocodonlithophilus*	CWH103	KF202303	KF202296
	CWH89	KF202302	KF202295
*Petrocodonviridescens*	Y .M.Shui et al 82661	HQ632939	HQ633036
	CWH41	KF202304	KF202297
*Petrocodonintegrifolius*	M.Moeller MMO 06-865	HQ632940	HQ633037
*Petrocodonlui*	Y .G.Wei 8012	HQ632938	HQ633035
*Petrocodontiandengensis*	09413	JX506850	JX506960
*Petrocodonainsliifolius*	Y .M.Shui et al 44071	HQ632941	HQ633038
	CWH88	KF202298	KF202291
*Petrocodonhispidus*	CWH101	KF202301	KF202294
	CWH87	KF202300	KF202293
*Petrocodonhunanensis*	WF190107-02	MK941180	MK941179
*Petrocodontongziensis*	Ren-Bo Zhang SBQ09383	MF872618	MF872617
*Petrocodonchishuiensis*	FW-2014	KF680503	KF680504
*Petrocodonwui*	WF065	OQ716553	OQ694978
*Petrocodoncoccineus*	CWH14B	KF202299	KF202292
	G80E	FJ501516	FJ501341
*Petrocodonhechiensis*	–	KR476563	KR337018
	M.Moeller MMO 07-1077	HQ632942	HQ633039
*Petrocodonhancei*	M.Moeller MMO 08-1342	HQ632944	HQ633041
	–	KC904959	KC904956
	–	KC904958	KC904955
	GDLC05	KF498253	KF498051
*Petrocodonasterocalyx*	FW-2013	KC904957	KC904954
*Petrocodonferrugineus*	M.Moeller MMO 06-784	HQ632946	HQ633043
*Petrocodonmultiflorus*	HJ01-2	KM232660	KJ475411
*Petrocodoncoriaceifolius*	M.Moeller MMO 06-913	HQ632943	HQ633040
*Petrocodonscopulorus*	W.Fang 2010-02	HQ632947	HQ633044
	–	GU350669	GU350637
	LJM06753	KR476567	KR337023
*Petrocodondealbatus*	LJM1209291	KR476565	KR337020
	G12B	FJ501537	JF697578
	LJM-2003-104	GU350668	GU350636

Note. “–” indicates that the author did not provide the voucher number.

## ﻿Taxonomic treatment

### ﻿ Petrocodon wui

Taxon classificationPlantaeLamialesGesneriaceae

F.Wen & R.B.Zhang sp. nov.

EB4C6CCE-5C83-552C-AC64-C1479E89166A

urn:lsid:ipni.org:names:77317789-1

Figs 1 , 2

#### Diagnosis.

*Petrocodonwui* is distinguishable by the elongated rhizome, the shape, size and indumentum of calyx lobes, the two conspicuous rows of orange glands on throat and the abaxial surface of the corolla lip. It morphologically resembles *P.chishuiensis*, but can be distinguished by having an elongated rhizome up to 30 cm or longer after years of growth (vs. lacking obvious rhizome in *P.chishuiensis*, following same order); leaf blade oval-oblong (vs. oblong or oblanceolate) and margin conspicuously undulate and densely ciliate (vs. serrate); cyme 4–10-flowered or more (vs. usually 1–3-flowered); anthers sparsely semi-transparent glands (vs. glabrous) and staminodes pale purple, club-like, glabrous (vs. absent or indistinctive).

**Figure 1.**
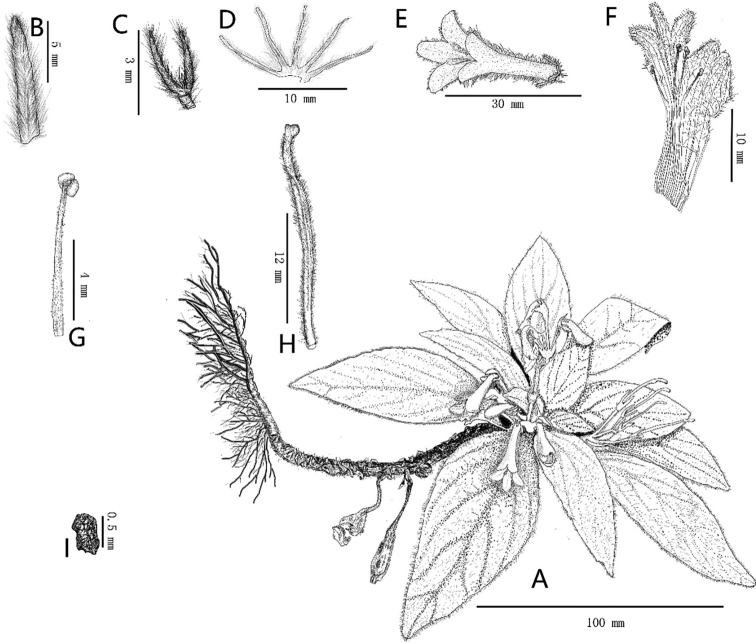
*Petrocodonwui* F.Wen & R.B.Zhang, sp. nov. **A** habit **B** bracts, showing the abaxial surface **C** bracteoles **D** abaxial surfaces of calyx lobe **E** top view of flower **F** opened corolla from the dried flower **G** one of stamens **H** pistil **I** seed. Drawings by Tan Deng from the type specimen.

#### Type.

China, Guizhou Province, Zunyi City, Xishui County, Xishui National Nature Reserve, Dabaitang, Niuqingshan, elev. ca. 1100 m, growing on a shaded and moist rock surface on the Danxia cliff in the gorge, *Ren-Bo Zhang ZRB2401* (holotype: IBK!, isotypes: ZY!).

#### Description.

Perennial herb, strictly lithophytic. ***Rhizome*** brown, abundant fibrous roots, especially at the nodes, rhizome becoming very long and up to 30 cm or longer after years of growth, the lower half of long rhizome usually growing downwards along the surface of rock with lots of fibrous roots, apex of rhizome usually curved and forming a hooked shape, some persistent base of petioles spirally arranged on the surface of rhizome; upper rhizome densely covered with villous multicellular hairs ca. 2 mm long with 4‒6 cells. ***Leaves*** in whorls of three, 6–15 crowded in a basal rosette or clustered at the top of elongated rhizome after years of growth, but usually some dried leaves persistent below foliage; petiole green, up to ca. 4 cm long, cylindrical, densely white pubescent; leaf blade chartaceous and thinly coriaceous when dried, oval-oblong, 6‒10 × 1‒3 cm, apex obtuse to acute or subacute, base cuneate, margin entire to inconspicuously or conspicuously undulate and densely ciliate, both surfaces densely white pubescent, lateral veins 4‒5-paired; ***Inflorescences*** 1‒4 or more, axillary, cymose, 4–10-flowered or more; peduncle pale green, 1‒4 cm long, ca. 1.5 mm in diameter, densely white villous; bracts 2, opposite, pale green, lanceolate, ca. 10 × 0.5 mm, apex acute, margin entire, both surfaces densely covered with villous multicellular hairs, ca. 1.5 mm long with ca. 3 cells; bracteoles 2, pale green, opposite, narrowly lanceolate, ca. 3 × 0.25 mm, indumentum same as bracts, but hairs on only ca. 2 cells; pedicels pale green, 0.8‒2 cm long, indumentum same as peduncle. ***Calyx*** 5-sected to near the base, but base slightly united forming calyx tube ca. 1 mm long; lobes equal, pale green to whitish-green, nearly linear, 6‒8 mm long, 5‒6 mm wide at the base, apex obtuse to rounded, margin entire, outside densely covered with white villous hairs, inside sparsely covered with white villous hairs. ***Corolla*** tubular, white, zygomorphic, ca. 2.5 cm long, outside densely white pubescent, inside nearly glabrous, upper part of corolla close to mouth puberulent; corolla tube 1.7–2.2 cm long, ca. 2.5 mm wide at the base of corolla tube and ca. 4.5 mm at the widest part of corolla tube; limb 2-lipped, adaxial lip shorter, 2-lobed to the middle, lobes broadly triangular, ca. 1.5 mm long, ca. 2.5 mm at the bottom of lobe, abaxial lip longer, 3-lobed to the middle or slightly exceeding the middle, lobes ovate, central one longer than lateral ones, ca. 3.5 mm long, lateral ones ca. 2.8 mm long, with two conspicuous rows of orange glands on abaxial lip and corolla throat. ***Stamens*** 4, two longer ones adnate to corolla tube ca. 9.5 mm from the base, filaments ca. 4.5 mm long, two shorter ones adnate to corolla tube ca. 8.5 mm from the base, filaments ca. 4 mm long, all filaments linear, straight, but slightly arched at the base and turning into a sheet at the base, white to semi-transparent, densely with brownish-black glands, especially from the middle to the base and glandular-puberulent close to the upper of filament; anthers brownish-purple to dark purple, dorsi-fixed, elliptic to nearly rounded, ca. 1 mm long, ca. 0.9 mm wide, coherent in pairs, thecae confluent at middle, sparsely semi-transparent glands, dehiscing longitudinally. Staminode 1, pale purple, club-like, glabrous, adnate to corolla tube ca. 8 mm from the base. Disc annular, ca. 1 mm high, margin entire. ***Pistil*** ca. 2.5 cm long, densely erectly glandular-pubescent; ovary linear-cylindrical, ca. 2 cm long, ca. 1.5 mm wide, 1-loculed, placentas 2, parietal; style ca. 6 mm long, ca. 0.8 mm wide; stigma 2, lobes lamellar, rounded to shallowly spatulate, glabrous, ca. 1 mm long, 0.9‒1 mm wide. ***Fruit*** a ***capsule***, ca. 5.5 cm long, linear-cylindrical, 4-valved, pubescent. ***Seeds*** appendaged, grain shortly cylindrical, rough, ca. 0.5 mm long, ca. 0.3 mm wide, covered densely verrucate.

#### Phenology.

Flowering occurs in August in the wild; fruiting should occur in October, based on current flowering patterns.

#### Etymology.

We dedicate this new species of *Petrocodon* to Wu Zheng-Yi (Wu Chengyih) (1916–2013), who devoted over 70 years to the flora of China. The scientific name, “*wui*”, is the latinisation of Wu Zheng-Yi’s family name. Coincidentally, a plant enthusiast, Lady Xiang-Hong Wu, took this species’ flowering photos in 2017 and sent them to one of the authors (Fang Wen) and her surname is also Wu.

#### Vernacular name.

The Chinese name proposed here is “吴氏石山苣苔.” Phonetically, it is “Wú Shì Shí Shān Jù Tái”.

#### Distribution and ecology.

The new species is endemic to Guizhou Province and known only from the type locality, Xishui National Nature Reserve in Xishui County. It grows on the steep Danxia cliff in an evergreen, broad-leaved forest in a valley of the Danxia landform, at an altitude of 1100‒1600 m. The cliff slope faces northwest at an angle of up to 60 to 80 degrees. The tree cover is up to 12 m tall, the canopy cover is 75%, the shrub layer cover is 85% and the herb layer cover is 35%.

#### Conservation status.

*Petrocodonwui* is known only from the type locality, which is protected by national and local laws and regulations. However, it is clearly scarce, being known from only one very small area of occupancy, estimated at 20 m^2^ on a rock surface in a valley of the Danxia landform. Obviously, this area of occupancy of *P.wui* we found so far is significantly lower than the smallest AOO unit of IUCN which is 4 km^2^ (2 × 2 km^2^ grid) for Critically Endangered B2. According to the detailed information from our careful field observations on the surroundings of the type area, the known population has about 50 individuals, half of those being mature individuals and half being seedlings. According to the Guidelines for using the IUCN Red List Categories and Criteria (IUCN Standards and Petitions Committee 2022), *P.wui* is provisionally assessed as “Critically Endangered, CR B2ab(ii) + C2b” because of its limited distribution and vulnerable habitat.

#### Taxonomic and phylogenetic notes.

**Figure 2.**
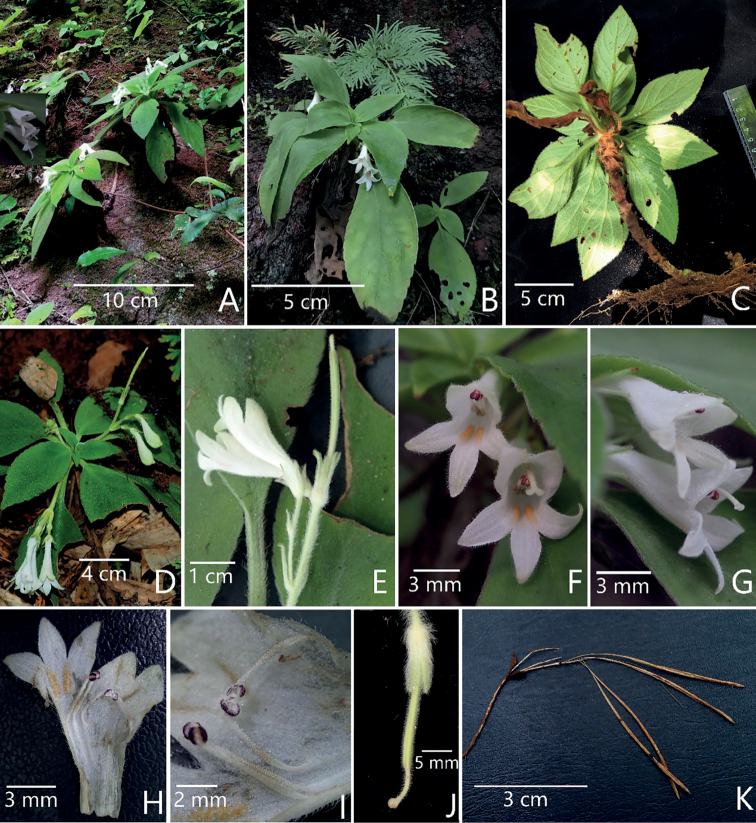
*Petrocodonwui* F.Wen & R.B.Zhang, sp. nov. **A** plants in bloom in natural habitat **B** plant in flower **C** upward view of plant showing the abaxial surfaces of leaf blade and petiole **D** flowering cyme **E** cymes, calyx and immature capsule **F** frontal view of corolla **G** lateral view of corolla and extended pistil **H** opened corolla **I** four fertile stamens **J** calyx and pistil **K** mature and dehiscent capsules (Photographed by F. Wen and R.B. Zhang).

**Figure 3.**
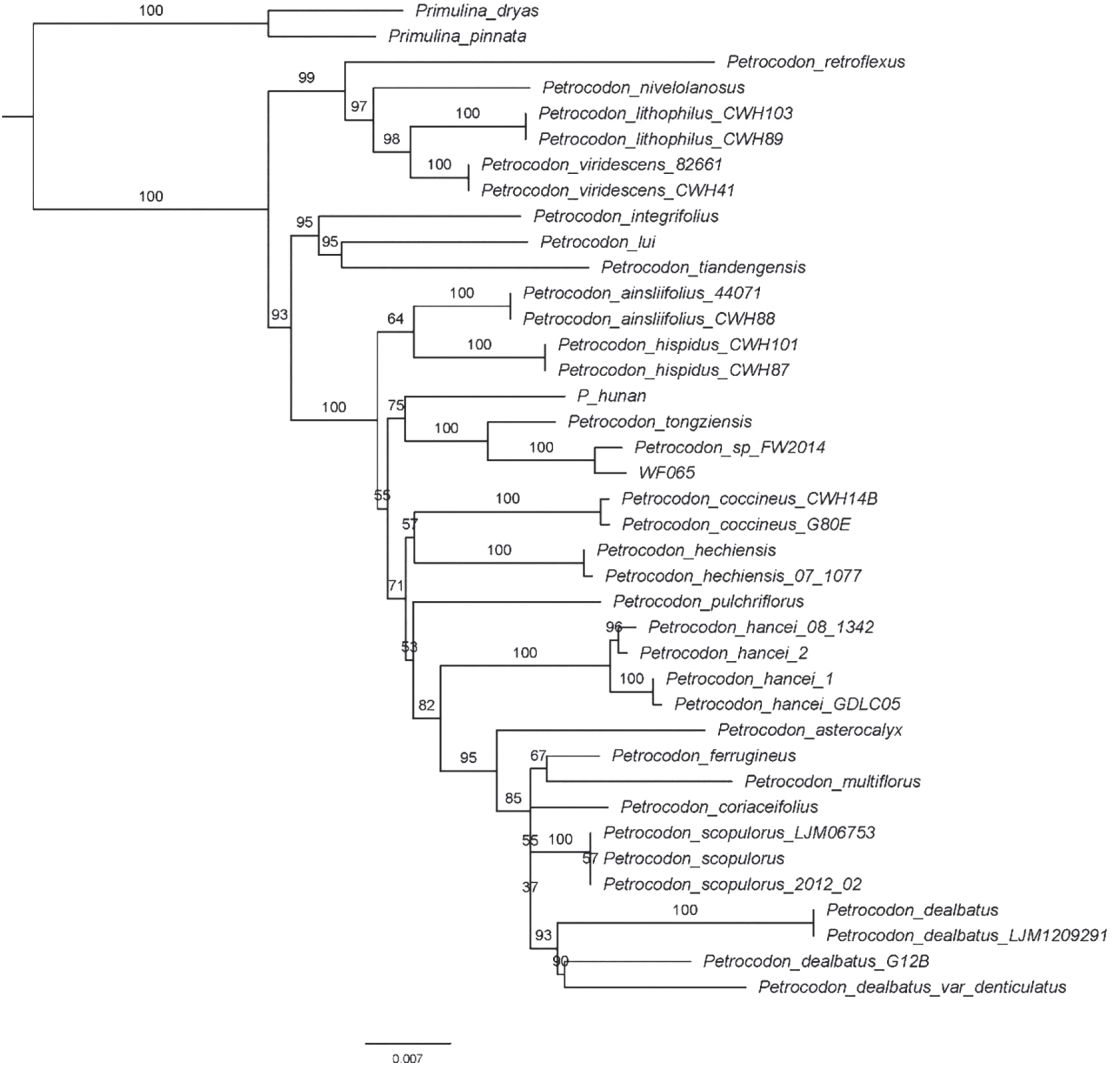
Phylogenetic tree of *Petrocodon* generated from Maximum Likelihood (ML) of *trnL-F* and ITS datasets. Numbers on the branches indicate ML bootstrap values (≥ 50%).

The aligned matrix of *trnL-F* and ITS sequences comprised 1594 characters. Of the 370 (23.21%) variable characters, 222 (13.93%) were parsimony informative. The phylogenetic trees revealed that all sampled *Petrocodon* taxa clustered together as a monophyletic group (BP = 100%), which is consistent with previous studies (Yang et al. 2022). Three strongly-supported clades are attributed to *Petrocodon*. Of these, the new species belonged in a moderately-supported subclade (BP = 75%) that also includes *P.hunanensis* X.L.Yu & Ming Li (Weber et al. 2011), *P.tongziensis* R.B.Zhang & F.Wen and *P.chishuiensis* (*Petrocodon_sp_FW2014*) (Fig. 3). This clade, denoted in Zhang et al. (2019), has four fertile stamens as a synapomorphy and our morphological observation of the new species supported this (Fig. 2). Within this clade, the new species is most closely related to *P.chishuiensis* (BP = 100%) (Fig. 3), whereas it can be easily distinguished from the latter by its rhizome, leaf blade, flowers number per cyme, bracts, bracteoles, calyx, filaments, anthers and staminodes, all of which are presented in Table 2.

**Table 2.** Morphological comparison of *Petrocodonwui* and *P.chishuiensis*.

Characters		*P.wui*	*P.chishuiensis*
Rhizome		present, becoming very long and up to 30 cm or longer after years of growth	lacking rhizome
Leaf blade			
	Shape	oval-oblong	oblong or oblanceolate
	Margin	entire to inconspicuously or conspicuously undulate and densely ciliate	serrate
	lateral veins	4‒5-paired	5‒6-paired
Flowers number per cyme		4–10-flowered or more	usually 1–3-flowered
Bracts			
	Shape	lanceolate	oblong
	Width	ca. 0.5 mm wide	ca. 3 mm wide
	Indumentum	both surfaces densely covered multicellular nodose villous and hairs ca. 1.5 mm long with ca. 3 cells	outside whitish pubescent, inside sparsely pubescent
Bracteoles	Size	ca. 3 × 0.25 mm	6–7 × ca. 1.5 mm
Calyx		5-sected to near the base, but base slightly united forming calyx tube and tube ca. 1 mm long	5-sected from base
Filaments			
	Length	two longer ones 4.5 mm long, two shorter ones ca. 4 mm long	two longer ones ca. 9 mm long, two shorter ones ca. 8 mm long
	Indumentum	densely with brownish-black glands especially from the middle to the base and glandular-puberulent close to the upper of filament	densely with glandular-puberulent hairs especially at the base
Anthers			
	Length	ca. 1 mm long	ca. 1.8 mm long
	Indumentum	sparsely semi-transparent glands	glabrous
Staminodes		pale purple, club-like, glabrous	absent or extremely indistinctive

## Supplementary Material

XML Treatment for Petrocodon wui
